# A conceptual framework for self-advocacy by people with intellectual disabilities

**DOI:** 10.4102/ajod.v14i0.1594

**Published:** 2025-05-29

**Authors:** Babalwa P. Tyabashe-Phume, Sharon R. Kleintjes

**Affiliations:** 1 Department of Psychiatry and Mental Health, Faculty of Health Sciences, University of Cape Town, Cape Town, South Africa; 2 Department of Global Health, Faculty of Medicine and Health Sciences, Stellenbosch University, Cape Town, South Africa

**Keywords:** self-advocacy, intellectual disability, conceptual framework, empowerment, Ubuntu, policy participation

## Abstract

**Background:**

People with intellectual disabilities are generally not consulted in the development of public policies, which impact their lives, and little is known about how to best empower people with intellectual disabilities to enable them to participate in public policy processes.

**Objectives:**

Our article reports on developing a conceptual framework to support self-advocacy by people with intellectual disabilities in social and health-related policy development in South Africa.

**Method:**

Our qualitative study was conducted using empowerment theory and integrated the concept of Ubuntu as a guide and was underpinned by a phenomenological approach. Data were collected through a scoping review, semi-structured interviews and focus groups. The scoping review was conducted using the Joanna Briggs Institute (JBI) scoping review protocol. Semi-structured interviews and focus groups were analysed using framework analysis. Data sources were triangulated to develop the conceptual framework, using a process adapted from three approaches used to develop similar conceptual frameworks.

**Results:**

Data triangulation identified three core elements for self-advocacy: (1) personal development; (2) creating a supportive environment to facilitate the empowerment of people with intellectual disability; and (3) improved policy participation opportunities.

**Conclusion:**

Participation of people with intellectual disabilities in public policy decisions, which can improve their quality of life, can be supported by developing their capacity for participation and increasing policymakers’ understanding as well as facilitation of what is needed to support their participation.

**Contribution:**

Our study offers a framework for a comprehensive approach to supporting people with intellectual disabilities in participating in and influencing public policy processes that impact their lives.

## Introduction

Self-advocacy refers to the ability and skills to communicate verbally, in writing, using pictures or gestures on behalf of oneself to motivate for the meeting of one’s needs (Mallander et al. 2018; Petri, Beadle-Brown & Bradshaw 2017). Self-advocacy is particularly important for people with intellectual disabilities who have historically been excluded from contributing to issues important to them (Goldberg & Kleintjes 2022). Different degrees of and sites for self-advocacy may be possible depending on a person’s capacity for participation and support available for this participation (Goldberg 2019; Kleintjes, Lund & Swartz 2013). Self-advocacy can occur at the level of influencing others to have regard for one’s in-home choice-making, through to choice-making in school, neighbourhoods and occupational settings. This includes broader societal-level choice-making in civil society actions such as policy participation (Petri et al. 2017).

Current principles and practices support the position that people with intellectual disabilities should enjoy a democratic right to self-representation in public policy development and implementation (Capri et al. 2018; Petri et al. 2017). However, people with intellectual disabilities still face restrictions in speaking up for themselves and standing up for their rights (Capri et al. 2018). Supporting them to express their opinions in public policy processes requires reasonable accommodations to account for participation restrictions that may be experienced because of cognitive and adaptive functioning impairments, as well as concerted efforts to remove existing discriminatory policy, organisational, procedural and practical barriers to participation (Kleintjes et al. 2013).

Self-advocacy by people with intellectual disability was spearheaded by parents and individuals with developmental disabilities internationally during the 1970s (Krueger, Van Heumen & Van Den Helder 2024). Over the years, the self-advocacy movement has grown globally, and many countries are moving in that direction, with the literature indicating that self-advocacy initiatives are more prominent in middle- and high-income countries (Henderson & Bigby 2016; Krueger et al. 2024). This is not to say that there are no challenges in these countries. In the United Kingdom (UK), for example, self-advocacy movements have been negatively impacted by austerity measures, with budget cuts, including the reduction of funding of self-advocacy organisations (Rouse et al. 2022).

Many people with intellectual disabilities and their families in South Africa do not have adequate access to health, social and other related public services. There is also a notable gap in service provision between rural and urban areas in South Africa, with the latter receiving higher-quality services. In particular, assistance for people with intellectual disabilities who exhibit challenging behaviours is inadequate. Where community-based services are available, they are primarily run by non-governmental organisations (NGOs), which also face several barriers to providing quality services, including funding constraints (Daniels 2018).

Advocacy by service providers and family members to address policy and service gaps is important. However, self-representation is a powerful tool to persuade policymakers and implementers to address the needs of citizens with intellectual disabilities. Goldberg (2019) argues that people with intellectual disabilities’ participation in self-advocacy at the level of public policy is supportive of their constitutional rights as citizens. This can provide them with opportunities to be heard in public processes and lessen the stigma that policymakers have towards them by challenging their views through exposing policymakers to these citizens’ contributions to the policy landscape.

There is a strong disability lobby that supports the participation of people with disabilities in public policy processes, in particular for people with physical disabilities. There is some movement for the inclusion of people with psychosocial disabilities in policy-level engagements, but self-advocacy by people with intellectual disabilities is still lacking (Kahonde 2023; Kleintjes et al. 2020). Small self-advocacy initiatives that include people with intellectual disabilities have been established in a few of South Africa’s nine provinces, with the assistance of NGOs, in particular, the Western Cape Forum for Intellectual Disability (WCFID) and the South African Federation for Mental Health (SAFMH) and some of its branches. The latter’s programmes aim to improve people’s ability to lobby, self-advocate and actively participate in initiatives that impact them. This also provides opportunities for individuals to comment on legislative, policy and service delivery developments, as well as address human rights violations.

This work remains largely unfunded outside of meagre budgets raised by these NGOs and falls short of the necessary capacity to be a strong, cohesive and representative voice for people with intellectual disabilities. There is still a significant need for active work to ensure that local intellectual disability organisations are sustainably developed, including self-advocacy groups. Ideally, the involvement of people with intellectual disabilities and their families must be strengthened, expanding self-advocacy to promote inclusion and access to societal resources (Anderson & Bigby 2017).

There have been attempts to provide a conceptual understanding of the actions needed to promote the participation of people with intellectual disabilities in various aspects of society (Tyabashe-Phume, Nkala-Shongwe & Kleintjes 2024). No local or international frameworks were found at the time of writing to guide a comprehensive response to empowering people with intellectual disabilities and their supporters to improve their participation in policy development. This article is derived from the first author’s PhD thesis (Tyabashe-Phume 2023), which aimed to develop a conceptual framework to guide self-advocacy for people with intellectual disabilities within a policy and service development context in South Africa. We present the process of development and components of a conceptual framework for self-advocacy by people with intellectual disabilities in the service development and policy domain in this article.

## Methods

Our qualitative study took a phenomenological approach to exploring the lived experiences of participants to gain deeper insights into their views on the focus of our study. This approach was most suitable to conceptualise, through interpretation of the data, the core elements of a support system required to support self-advocacy efforts by people with intellectual disabilities.

### Data collection to inform the framework’s elements

We collected data through a scoping review, semi-structured interviews (SSIs) and focused group discussions. The purpose of the scoping review was to identify key concepts relevant to supporting people with intellectual disabilities’ direct policy participation. The scoping review also aimed to identify the existing conceptual frameworks for self-advocacy, which could inform the methodology for developing the conceptual framework. By following Joanna Briggs Institute’s Preferred Reporting Items for Systematic reviews and Meta-Analyses extension for Scoping Reviews (Peters et al. 2020), an initial 168 articles were identified after a rigorous inclusion and exclusion process, with 20 articles finally included for review (Tyabashe-Phume et al. 2024). Twenty-five SSIs were conducted with five groups of stakeholders, including people with intellectual disability (*n* = 5), their parents (*n* = 5), carers or supporters (*n* = 5), policymakers (*n* = 5) and service providers or managers (*n* = 5). The SSIs were aimed at exploring these stakeholders’ views about the need for self-advocacy by people with intellectual disabilities, opportunities for and barriers to self-advocacy and strategies to overcome these barriers.

Box 1 details the selection requirements for participation of people with intellectual disabilities. The study participants were limited to individuals who had certain verbal, social and cognitive capabilities and some understanding and experience of self-advocacy.

BOX 1 Eligibility of participants with intellectual disabilities.

- Older than 18 years of age
- Able to understand research aims and purpose of the interviews
- Able to communicate verbally in English, Afrikaans or IsiXhosa
- Willing to participate in individual and/or focus group interviews
- Not experiencing mental or emotional distress at time of interview.
- Able to participate voluntarily and aware withdrawal was allowed
- Has some experience of involvement in self-advocacy work

Because of strict coronavirus disease 2019 (COVID-19) regulations in place at the time of our study, all interviews were conducted online via Zoom and WhatsApp calls. Easy-to-read information sheets and consent forms, and assistance by a supporter well known to them, were put in place as the means of reasonable accommodation.

### Data analysis

#### Scoping review

Two authors independently extracted data from 2 of the 20 included articles and then compared and finalised the extraction process, after which the first author extracted data from the rest of the article. Relevant scoping review data were extracted to forms for both quantitative and qualitative studies using Population, Intervention, Comparison, Outcome, Study (PICOS) design and Sample, Phenomenon of Interest, Design, Evaluation, Research (SPIDER) type, respectively (Cooke, Smith & Booth 2012).

#### Semi-structured interviews

Interview data were analysed with the aid of the Atlas.ti software (version 9.1.7), using framework analysis, a structured form of thematic analysis that identifies similarities and differences in qualitative data as well as connections between various data elements in order to derive themes, subthemes and conclusions that are either descriptive or explanatory in an iterative process (Gale et al. 2013; Ritchie & Spencer 2002).

#### Data triangulation

The process of triangulating the data was derived from three frameworks. The first framework, sourced during an initial overview of the literature, has been used to strengthen the participation of individuals with psychosocial disabilities and carer involvement in the National Institute for Mental Health England (NIMHE), an organisation affiliated with the National Health Service in the UK (HASCAS 2005). The scoping review found two further frameworks: Cook’s multicultural and social justice counselling competences framework (Cook 2017) and Test and colleagues’ self-advocacy framework (Test et al. 2005). The methods employed to create these three frameworks were considered while determining the method to develop our study’s conceptual framework. Table 1 summarises the processes used in the three frameworks and their adaptation for use in our study.

**TABLE 1:** Guiding frameworks.

Process of developing a conceptual framework	Reviewed frameworks		
	HASCAS (2005)	Test et al. (2005)	MCSJCC (Cook 2017)
Scoping review to identify existing conceptual frameworks for self-advocacy and principles and strategies to support participation, as well as to assess legislative policy and stakeholder support for participation	Literature scan to identify key issues, principles and good practice. Reviewing NIMHE strategy about service user and carer involvement.	Extensive literature review of 20 articles pertaining to self-advocacy	Review of the MCC framework that was developed by Sue et al. (1992) and review of similar literature from other professions regarding the MCC
Semi-structured interviews to identify stakeholder understanding of self-advocacy, views on the need for self-advocacy, barriers and opportunities for self-advocacy and support for participation by people with intellectual disability	NIMHE stakeholder interviews on user or carer involvement strengths and difficulties, questionnaire on NIMHE involvement arrangements, focus groups with users and carers to explore the issues raised above, attending meetings of NIMHE networks and MindLink mailing list questionnaires to service users and carers	Engagement of relevant stakeholders	Engagement of various stakeholders and associations to adapt and update the MCC framework to the MSJCC framework
Researcher triangulation of all data sources for the first draft of the conceptual framework. Translation of framework to the easy-to-read format by Cape Mental Health’s easy-to-read consultation group of advisers with intellectual disability and supporters.	Drafting of the framework with the assistance of user and carer reference group and expert consultation	-	-
Consultation to finalise draft conceptual framework: Review of Draft 1 (two focus groups, two individual discussions and advisory board meeting), incorporation of feedback into final version of the framework	Consultation: Wide circulation of background reports and framework for 2 months, and incorporation of comments to finalise the framework	Engagement of stakeholders to provide feedback on the draft of the conceptual framework	Engagement of stakeholders to provide feedback on the framework

NIMHE, National Institute for Mental Health England; MSJCC, Multicultural and Social Justice Counseling Compitencies; MCC, Multicultural Counseling Competencies.

#### Drafting of the conceptual framework

Using the triangulated data, an initial draft of the elements comprising the conceptual framework was developed by the first author based on thematic categories derived from the findings, as elaborated hereunder.

#### Focused group discussions

These were conducted to better understand how our study participants felt or thought about the conceptual framework that has been developed (De Vos et al. 2011). Participants who had consented to critique the draft conceptual framework were invited to provide their feedback on whether the authors had accurately captured their views in interpreting the data they had provided. Two focus group discussions were convened. The first focus group discussion included policymakers and service managers (*n* = 5). The second group included parents and carers or supporters (*n* = 3). The conceptual framework was presented to the groups, and each element was explained in detail. Then, the participants were given an opportunity to ask questions and provide feedback.

The conceptual framework was translated into an easy-to-read document for participants with intellectual disabilities. Although the review with individuals with intellectual disabilities was initially planned to be a focus group, it was not feasible because of COVID-19 restrictions on face-to-face meetings, the need to use Zoom, and scheduling conflicts among participants. Consequently, the review was conducted through two individual discussions with participants with intellectual disabilities with the support of their support personnel.

### Ethical considerations

An application for full ethical approval was made to the University of Cape Town’s Faculty of Health Sciences Research Ethics Committee, and ethics consent was received (HREC No. 019/2021). Permission was also sought and received from three government departments (Departments of Health, Basic Education and Higher Education and Training) to conduct interviews with officials in those departments. Written informed consent was obtained from all individual participants involved.

## Results

Our findings yielded five main thematic categories during an iterative analysis process. These categories were instrumental in identifying and formulating the essential elements of the conceptual framework designed to support self-advocacy by people with intellectual disabilities in the context of policy and service development in South Africa. Table 2 summarises the thematic categories elicited from the triangulated data sources.

**TABLE 2:** Thematic categories from the scoping review and semi-structured interview.

Thematic categories category	Data source		
	Scoping review	SSI with parents, supporters and people with ID	SSI with policymakers and service managers
Meaning of self-advocacy by people with intellectual disability	√	√	√
Ubuntu and Interdependence (supported self-advocacy)	√	√	√
Barriers to self-advocacy	√	√	√
Inclusive policy environments	√	√	
Capacity building	√	√	√

SSI, semi-structured interview; ID, intellectual disability.

### Meaning of self-advocacy

Self-advocacy was defined as the ability of people with intellectual disabilities to speak up for their rights, make informed decisions, and advocate for their needs. Findings emphasised the importance of skills such as self-confidence, communication, assertiveness, decision-making and public speaking. One participant with an intellectual disability, for example, stated that:

‘Self-advocacy actually means that I can talk for myself, I can protect myself, and I can stand up for myself! Because, you know, in the world, there are people who don’t regard people with disabilities as advocates or don’t seem as an advocate to them. So, I can actually tell them, no, I am a person, and I can talk for myself. No one can make decisions for me; I can make my own decisions.’ (Participant T2, female, self-advocate with intellectual disability)

Most participants emphasised that self-advocacy involves people with intellectual disabilities asserting their rights, including constitutional and human rights, challenging policies that undermine these rights, and advocating for greater inclusion and active participation in society. The interview findings aligned with the scoping review, emphasising that people with intellectual disabilities should be included in decision-making processes to voice their opinions on matters that impact them (Perkins 2010; Petri et al. 2017). Like all individuals, they possess equal rights, including the right to contribute to decisions that affect their lives:

‘Everyone within the country has a constitutional right to be heard and, and there’s a constitutional right to participate in society just like everyone else, more than anything … and self-advocacy allows people with disabilities to do that, allows people to participate just like everyone else.’ (Participant S3, male, service manager)

### Ubuntu and Interdependence (supported self-advocacy)

People with intellectual disabilities often rely on support from those around them. Some participants with intellectual disabilities expressed that developing self-advocacy skills requires assistance:

‘I would say, the kind of support that would help me to be able to advocate in front of the whole nation. Where I could stand in front of everyone and speak up … Some people like me need to learn to speak up for themselves. I also learnt to speak up from this programme (Self-advocacy group).’ (Participant T1, female, self-advocate with intellectual disability)

Participants identified several essential personal skills for people with intellectual disabilities to become self-advocates, including communication, assertiveness, confidence, public speaking and leadership, among others. Support systems, including peer mentoring, family involvement and professional assistance, were also noticed as playing a critical role in enabling self-advocacy. Findings highlighted the need for co-presentation of ideas, where supporters assist people with intellectual disabilities in expressing their views while ensuring their autonomy. Access to resources, tailored accommodations and existing opportunities, such as self-advocacy groups, were identified as pivotal. However, participants felt that there were limited opportunities for participation in South Africa, necessitating the creation of more inclusive programmes and platforms to promote active participation.

### Barriers to self-advocacy

Barriers to self-advocacy included individual challenges such as limited communication skills, societal stigma and structural exclusion from decision-making processes. Participants observed that people with intellectual disabilities are often infantilised or denied the opportunity to make decisions. Policymakers’ lack of knowledge and understanding further restricts inclusion. Supported decision-making, where individuals are guided without coercion, and advocacy by peers or supporters were suggested as solutions to overcome these barriers. Addressing discrimination and fostering respect for autonomy were also emphasised:

‘There’s a lot of stigma around being belittled. People expect you to follow them. Instead of asking me, what do you want, or don’t you want to or do you want me to decide for you?’ (Participant T2, female, self-advocate with intellectual disability)

### Inclusive policy environments

Inclusive policy environments require proactive efforts from policymakers, supporters and service providers to ensure the participation of people with intellectual disabilities. Current exclusionary practices were attributed to a lack of understanding of intellectual disabilities and inadequate accommodations. One policymaker stated:

‘I do think that professionals not really understanding intellectual disability and their needs is a problem when it comes to policy-making processes.’ (Participant Pol 5, female, policy maker)

Recommendations for creating supportive policy environments included providing accessible materials to people with intellectual disabilities, fostering peer-to-peer support and enabling voluntary participation. Participants emphasised that inclusive environments should empower individuals to express their needs freely and provide individualised support to facilitate meaningful engagement in policy processes.

### Capacity building

Several participants were of the view that capacity building is an essential element needed to support self-advocacy for people with intellectual disabilities. Key components identified were skill-building to enable meaningful self-advocacy, training and development for those supporting people with intellectual disabilities, and capacity building for policymakers and service managers to foster inclusive policy environments. Findings from the scoping review and interviews emphasise that training and the act of participating in policymaking initiatives and processes are needed to enhance self-advocacy abilities and to develop essential self-advocacy skills.

Policymakers and service managers also identified their own need to be educated about intellectual disabilities. They felt that training would equip them with the skills to effectively engage with individuals with intellectual disabilities and provide the necessary support during policymaking processes. They noticed that raising awareness about and offering guidance on implementing reasonable accommodations in policy environments could significantly improve the participation of individuals with intellectual disabilities.

During the focused group discussions, policymakers and service managers suggested using positive language in the framework. They emphasised the need for people with intellectual disabilities to understand their rights and policies. They also highlighted the importance of supporting self-advocacy and capacity building, as well as the need for funding to support self-advocacy programmes. Parents and supporters found the conceptual framework practical, although they did not propose changes. Some parents initially doubted their children’s ability to self-advocate in policy, but one parent gained confidence after seeing the support provided by the framework:

‘Like I said before and during our interview, we need to invite more people with intellectual disability to review policies and we need to consult them more. You know, their contributions can go a long way in developing policies. When we know what they have to say about their rights it will make our jobs easier, it will make things easy for everyone actually. Just like we invite people without disabilities to comment on policies, we should make sure that people with intellectual disability are invited as well, for representation, you know.’ (Participant Pol 1, female, policy maker)

Storytelling emerged as an important tool for people with intellectual disabilities to convey their policy input. This was also demonstrated during their feedback interviews. Participants with intellectual disabilities were supportive of the framework, elaborating on the framework components based on their lived experiences:

‘So, like, you know, the government decides that they will give us, us people with disability the SASSA grant. So, they say they will give people with disabilities a grant of this money, but now they do not ask us, like maybe to say how much money do we think we will need for the grant that we think will be enough. Like they just decide for us and it’s not nice, man.’ (Participant T4, male, self-advocate with intellectual disability)

Participants’ feedback was incorporated into the draft to arrive at the final version of the conceptual framework.

## The conceptual framework for self-advocacy

**FIGURE 1:**
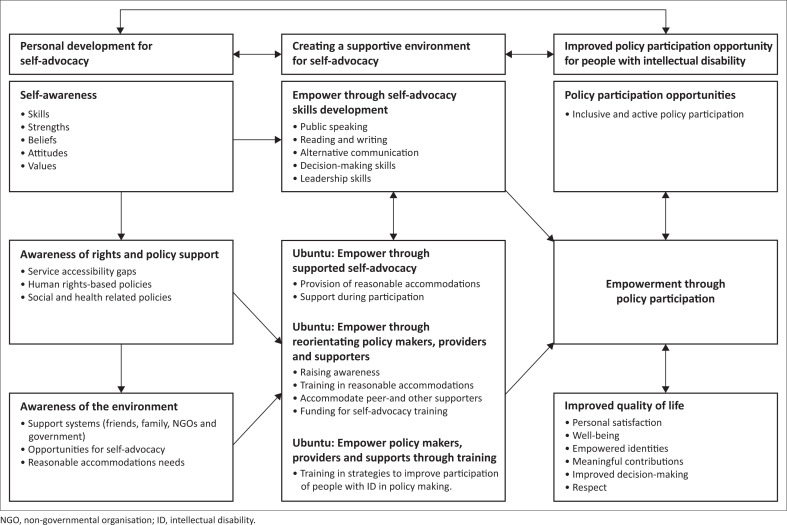
Conceptual framework for self-advocacy by people with intellectual disabilities.

The conceptual framework (Figure 1) was derived from strategies recommended for creating and improving policy participation by people with intellectual disabilities. It is grounded in the principles of the empowerment theory and the concept of Ubuntu.

The framework consists of three interconnected elements: Personal development for self-advocacy, creating a supportive environment for self-advocacy and improved policy participation for people with intellectual disabilities. The discussion begins with personal development and then progresses from left to right in Figure 1, explaining the other elements and how they interconnect.

### Personal development for self-advocacy

The primary focus of this element of the conceptual framework is drawn from participants’ views that people with intellectual disabilities will require self-development to facilitate their self-advocacy in policy processes. This element comprises three sub-elements: self-awareness, awareness of rights and policy support and awareness of the environment.

#### Self-awareness

This sub-element relates to the capacity to know who you are, your values, beliefs and biases. It emphasises exploring the existing personal, interpersonal and environmental abilities and skills of individuals with intellectual disabilities, as well as identifying areas that need further development to reach their personal goals. Self-awareness is vital for self-advocacy, as individuals must first understand themselves before they can effectively communicate their needs and desires to others.

#### Awareness of rights and policy support

The second sub-element focuses on the need for people with intellectual disabilities to know what their rights are and the extent to which policies address their rights. This is important for them to become aware of infringements of their rights, enabling them to make informed decisions about what they might want to advocate for in terms of rights infringements. It also assists in their using rights-based policies to self-advocate for their needs. Moreover, they need to recognise both their fulfilled and unmet service needs, understand the services they are entitled to and provide their views on whether they experience these services as adequately provided.

#### Awareness of the environment

The third sub-element relates to people with intellectual disabilities being aware of and knowing their environment and the world at large within their capabilities. This involves being aware of their support networks, such as family, friends and peers, as well as governmental structures and opportunities for self-advocacy within public sectors. In addition, it includes recognising other community-based support systems, like local organisations working with people with intellectual disabilities, which can serve as allies. Another important aspect of environmental familiarisation is exposure to these environments, as well as allocation of sufficient time and support to learn and gain confidence in engaging with others in different formal and informal social spaces in which they may wish to engage.

### Creating a supportive environment for self-advocacy

The second element of this conceptual framework focuses on how families, supporters, service managers, providers and policymakers can assist people with intellectual disabilities in self-advocacy.

The primary contention of this element is that the empowerment of people with intellectual disabilities to self-advocate in policy processes is most effectively done when they can rely on those around them when they wish to advocate for themselves. Supported self-advocacy recognises the mutual reliance between individuals with intellectual disabilities and their families, friends and other supporters. When people provide support to people with intellectual disabilities to make, carry out and experience the benefits of their own choices, it can boost their sense of being included, valued, autonomous, competent and self-directed (Anderson & Bigby 2023).

The understanding of the concept of interdependence as used in this research is best described in terms of ‘Ubuntu’, an African philosophy that emphasises ‘being self through others’. Ubuntu is a form of humanism, which can be expressed in the phrase *I am because of who we all are* (Mugumbate & Andrew 2013). Ubuntu holds that every person forms a link in a chain of human relationality, which should be reflected in the interconnectedness of people within society. This conceptual framework incorporates this philosophy of Ubuntu, observing that each person is embedded in reciprocal interactions, which can be leveraged for mutual support of each other to be their full selves, not overshadowing or dominating one or the other. Genuine support for people with intellectual disabilities in self-advocacy must prioritise their own perspectives rather than supporters or family members deciding what is best for them. Through the philosophy of Ubuntu, individuals with intellectual disabilities can gain empowerment by receiving assistance in areas where they face challenges to communicate their needs, choices and decisions.

Preparing people with intellectual disabilities for participation involves establishing supportive systems and engaging support personnel in this process (Roberts, Ju & Zhang 2016). These key individuals play an essential role in preparing people with intellectual disabilities for active involvement. Support systems include community members in both formal and informal positions of power, NGOs working with individuals with intellectual disabilities, and allies such as friends, peers and family. In line with human rights and social inclusion, these support persons and organisations are crucial in the South African community for facilitating the participation of people with intellectual disabilities in policy development and enabling self-representation or supported decision-making.

To effectively support people with intellectual disabilities, role-players must act within their organisations to create more inclusive policy spaces, enhance collaborations with existing disability structures and reinforce disability-led forums. People with intellectual disabilities and their allies should also increase their involvement with organisations such as the South African Human Rights Commission to challenge policies and practices that violate their rights. Additionally, they should engage with government departments responsible for overseeing policies and programmes related to women, children and people with disabilities to advocate for their needs and ensure their priorities are included in the government’s agenda.

The framework posits that for people with intellectual disabilities to participate more fully in self-advocacy, they require empowerment through two main sub-elements: (1) empowerment through self-advocacy skills development; and (2) empowerment through supported self-advocacy.

#### Empowerment through self-advocacy skills development

Self-advocacy skills-building can strengthen self-advocates’ sense of personal identity, help develop supportive friendships, enhance their sense of being part of a community of common interest and promote self-determination (Balint-Langel et al. 2020; Grove 2015). Self-advocacy skills can collectively be considered self-determination skills (Grove 2015). Empowerment in this regard, then, refers to supporting people with intellectual disabilities to learn practical skills to assist them in self-advocacy. Examples of these areas of skill that arose in our study include public speaking, reading and writing skills in alternative communication methods, decision-making skills and leadership skills. For people with intellectual disabilities who cannot read or write, supporters can play a valuable role as readers and scribes for their ideas. They can request that information be written in an easy-to-read format and learn to use alternative communication methods, such as using pictures, recording videos or audio, social media and story-sharing to convey their ideas and advocate for themselves.

While help can be requested from a supporter or family member to help think through their ideas so that they can make informed decisions about what they will choose to say or do as a self-advocate, decision-making skills are important to advance their ability to make self-directed decision-making over time. Additionally, leadership skills are important for self-advocates. They may, in becoming aware of the common needs of other people with intellectual disabilities, develop interest in speaking up both for themselves and for other people with intellectual disabilities who cannot speak for themselves, that is, to both self-advocate and to advocate on behalf of others.

#### Empowerment through supported self-advocacy

We have found that it is through Ubuntu that policymakers, service providers, supporters, family and friends can be encouraged to create a comfortable and inclusive policy environment. The results indicated that these stakeholders need reorientation to overcome outdated views and attitudes towards people with intellectual disabilities.

Stakeholders who participated in our study noticed a need for training of policymakers, service providers and supporters about intellectual disability and ways in which they can create inclusive policy environments that allow meaningful participation by people with intellectual disabilities, including what might constitute appropriate reasonable accommodations for people with intellectual disabilities.

### Policy participation opportunities for people with intellectual disabilities

The third element focuses on the need for a focused effort to ensure real opportunities for policy participation that allow for the inclusive involvement of people with intellectual disabilities. This participation should be personally life-enhancing. Participants’ responses highlighted that when people with intellectual disabilities are actively involved in policy participation, there will be a positive impact that can potentially improve their quality of life. Participants felt that they would experience feelings of being respected, of being treated with dignity, of experiencing personal satisfaction and empowered identities through the ability to voice their perspectives and make their own decisions, and through experiencing the conviction that their efforts contribute meaningfully to their lives and the lives of others with and without intellectual disabilities.

Participation should also result in measurable influence on policy outcomes. Their input should be reflected in the inclusion of their views in policies on what should support their rights and needs. In the medium to long term, policy reviews and impact studies should indicate meaningful improvements in these citizens’ lives. Ubuntu, in this regard, emphasises that policymakers need to accept that it is important for them to facilitate people with intellectual disabilities having access to peer and other support during policy participation. In addition, funding is required to develop self-advocacy training and to provide resources for people with intellectual disabilities and their supporters to prepare, attend and provide input to policy-making processes.

The framework shows that, at the political level, people with intellectual disabilities should be given meaningful – not token – opportunities to engage more actively in the democratic processes of the state. Participants, for example, observed that care should be taken to ensure that public invitations to consultation meetings to comment on drafts of new policies should include people with intellectual disabilities and their supporters and provide adequate time for pre-meeting preparation of comments. In addition, policymakers should provide accessible forms of policy communications, which self-advocates or supporters can utilise in preparing for participation as well as permitting the opportunity to submit commentary via alternative communications to the usual written commentary, for example, video or auditory formats.

## Discussion

Currently, South Africans with intellectual disabilities do not participate equally in democratic processes. For instance, they are prohibited from voting or standing for election because they are considered to be mentally incompetent (Capri & Swartz 2018; Combrinck 2014). They are thus denied political equality (Kleintjes et al. 2020). Goal 16 of the sustainable development goals (SDGs) calls for non-discriminatory laws and policies that are inclusive of people with disabilities (United Nations 2016). Our results propose methods through which people with intellectual disabilities can be prepared for and supported to participate in electoral and other political and policy-related processes to have their voices heard.

In South Africa, NGOs supporting people with intellectual disabilities to self-advocate are still limited, with the key organisations being the WCFID as well as the SAFMH. Work to support self-advocacy initiatives are variously developed in these organisations at the time of writing this article. The history of people with intellectual disabilities organising for self-advocacy elsewhere strongly suggests that the best practice to support and capacitate people with intellectual disabilities in South Africa would be to consider expanding current efforts to also support these self-advocates to spearhead their own self-advocacy movements (Ledger & Tilley 2006).

Experiences from countries with established self-advocacy organisations indicate that transitioning to more formal structures can help people with intellectual disabilities feel a sense of ownership over self-advocacy efforts as experts by experience. This would involve a collective effort to collaborate with people with intellectual disabilities to mobilise to form their own self-advocacy groups with the support of their allies. However, this approach must consider challenges faced by current self-advocacy organisations, such as securing funding and providing adequate support to experts by experience within these groups (Ledger & Tilley 2006; Rouse et al. 2022). Further research would assist in addressing this for local implementation.

As suggested in the results and supported by the literature (Anderson & Bigby 2017, 2023), policy participation can promote feelings of self-efficacy and well-being through the ability to voice their perspectives and make their own decisions and through experiencing the conviction that their efforts contribute meaningfully to their lives and the lives of others with intellectual disabilities. With active policy participation, there can be a shift in the inclusion of people with intellectual disabilities (Kleintjes et al. 2013). This shift is central to enabling people with intellectual disabilities to move from being observers in deliberations about their lives to central, valued role-players within the policy development environment.

### Limitations

It is important to observe that the participants with intellectual disabilities in our study are not representative of all people with intellectual disabilities but rather were limited to people who are already self-advocates who are able to speak up for themselves on their own or with support and other accommodations. The resultant conceptual framework does not consider strategies for inclusion and participation for people with intellectual disabilities who have very high support needs and very limited capacity for policy influence. A limitation of the framework, then, is that it does not explore what is feasible to include for people with high support needs in efforts to advocate for their rights or co-advocate along with their supporters, namely families, service providers and other people with intellectual disabilities who do more readily have the capacity for self-advocacy. At present, in a South African context, people with greater degrees of intellectual disabilities are included by family and professional advocates in their advocacy efforts, the power of their very presence in political and policy spaces giving greater credence to families’ and service providers’ advocacy on their behalf.

## Conclusion

We aimed to develop a conceptual framework for self-advocacy by people with intellectual disabilities to engage in social and health-related policy-making and prioritising in South Africa. The framework emphasises the requirements for people with intellectual disabilities to engage in self-advocacy for their policy needs. It also identifies practical steps needed to ensure their active inclusion in policy processes. The framework breaks down the necessary conditions for individuals to thrive as self-advocates. It highlights the actions required by policymakers, service providers and supporters to foster an inclusive policy environment. Furthermore, it highlights the importance of focused efforts to ensure policy participation opportunities that allow people with intellectual disabilities to participate inclusively.

We emphasise that, despite contextual differences, the disempowerment, exclusion and the fight for empowerment and respect for their rights are universally shared by people with intellectual disabilities, including in South Africa. To enable policy participation, barriers should be removed and more inclusive environments should be created. Increased opportunities should be provided for people with intellectual disabilities to engage in the development of policies and services that enhance their well-being.
